# *Ganoderma lucidum* beta 1,3/1,6 glucan as an immunomodulator in inflammation induced by a high-cholesterol diet

**DOI:** 10.1186/s12906-016-1476-3

**Published:** 2016-12-03

**Authors:** Yu-Sheng Wu, Shu-Ying Ho, Fan-Hua Nan, Shiu-Nan Chen

**Affiliations:** 1 College of Life Science, National Taiwan University, No.1, Sec. 4, Roosevelt Rd., Da’ an Dist., Taipei City, 10617 Taiwan; 2 Department of Aquaculture, National Taiwan Ocean University, Keelung, 20248 Taiwan

## Abstract

**Background:**

Binding of beta 1,3/1,6 glucan of *Ganoderma lucidum* (*G. lucidum*) with the receptor results in a series of signal transfers (signalling cascades), which activates the transcription factors for regulating inflammation. Excess cholesterol intake leads to an increase in the distance between fat cells and capillaries, which may cause hypoxia in the fat tissue of obese mice. This hypoxia induces the death of fat cells, resulting in the inflammation of adipose tissue or an increase in the inflammatory gene expression associated with obesity.

**Methods:**

The current study examined the immunomodulation effect of *G. lucidum* beta 1,3/1,6 glucan according to immunoglobulin, poly-Ig receptor expression, Nature Killer cell (NK cell) activity, lymphocytes proliferation and cytokines expression.

**Results:**

Our present study shows that feeding *G. lucidum* beta 1,3/1,6 glucan to mice induces IgA or IgG expression in the serum and small intestine washing fluid and enhances poly-Ig receptor expression in the small intestine moreover, the observation of the IL-2 and Nature killer cell activity were exchanged.

**Conclusions:**

The effect of a high-cholesterol diet in the inflammatory response was observed in heart, liver, kidney, spleen, and colon tissues through histopathological evaluations. The presented evidence demonstrates that the inflammation response in the high-cholesterol diet group was much higher than in the other groups and the beta 1,3/1,6 glucan reduces inflammation in obese mice fed a high-cholesterol diet.

## Background

Previous studies have shown that dietary cholesterol affects the physiological performance of cells. The literature indicates that in patients with high cholesterol, endothelial cells, macrophages, and smooth muscle cell membranes have increased cholesterol content and undergo alteration in the membrane composition, which affects cell functions such as antigen-presenting capability and the interaction of monocytes and endothelial cells [1, 2]. *Ganoderma lucidum* is a medicinal mushroom which has been widely used in the China and Japan for hundreds of years for the immunomodulating, anti-inflammation and anti-tumor effects. Many biological available substances, in particular polysaccharides, with immunity enhancement effects have been isolated from the extract of *G. lucidum* [3]. Previous, it has found that the fruiting body extracts from *Lentinus edodes*, *Trametes versicolor*, *Ganoderma tsugae*, *Flammulina velutiper, Ganoderma lucidum* and *Tricholoma matsutake* demonstrated significant anti-tumor activities towards transplanted tumor cells of Sarcoma 180 [4, 5]. In previous studies, *Autrodia comphorata*-derived beta-glucan has demonstrated inhibitory effects on tumor growth for Sarcoma 37, Sarcoma 180, Erlich ascites sarcoma, Yoshida sarcoma and Lewis lung carcinoma-1 (LLC1) transplanted tumor growth [6].

Excessive intake of high-cholesterol foods leads to chronic inflammation, and altered expression of inflammatory genes and proteins including cytokines, chemokines, complement proteins, and adipokines has been observed in most adipose tissue [7]. Patients with a high body mass index usually present with higher concentrations of many adipokines, type 2 diabetes, and atherosclerosis [8]. Researchers conducting systematic observations have demonstrated that the macrophages from peripheral tissue penetrate human and mouse adipose tissue to release inflammatory substances, including TNF-α and IL-6. In addition, a necrotic response of numerous fat cells was observed, and pathologic findings suggested that adipose tissue macrophages surrounded these necrotic cells [9, 10]. Previous research has indicated that the excessive intake of fat leads to an increased distance between fat cells and capillaries, and this may cause hypoxia in the fat tissues of obese mice. This hypoxia induces the death of fat cells, which results in the inflammation of adipose tissue or inflammatory gene expression associated with obesity known as ‘endoplasmic reticulum stress’ [11, 12].

The intestinal mucosal immune response is mounted by the mesenteric lymph nodes, and antigens from gut-associated lymphoid tissue activate the antigen-presenting system and enhance the inflammatory response. Evidence indicates that inflammation contributes to systemic metabolic dysfunction, which is associated with inflammation disorders [13]. A study showed that cytokines and pathogen-associated molecular patterns (PAMPs) costimulate cell-surface receptors including Toll-like receptors (TLRs) to initiate intracellular signalling leading to the activation of NF-κB. NF-κB activation was proposed to induce target gene expression to promote cellular proliferation and activate the immune response [14].

Research has illustrated that β-glucans are PAMP molecules that are recognised by pattern-recognition receptors, such as TLRs and NOD-like receptors, and activate transcription of proinflammatory genes [15]. Moreover, they exhibit immune stimulatory activity and enhance wound healing, particularly by increasing macrophage infiltration to injury sites and stimulating tissue regeneration [16, 17].

The present research was performed in two parts; in the first part, mice were administered mushroom beta-glucan (MBG) to detect immunoglobulin (Ig) A and G expression in the intestine and serum. Altered poly-Ig receptor gene expression in the intestinal tissue was also detected. By detecting Ig and poly-Ig receptor gene expression, we explored the immunomodulation function of *Ganoderma lucidum* beta 1,3/1,6 glucan. In the second part, we examined the regulation of the inflammatory response by *G. lucidum* beta 1,3/1,6 glucan through histopathological evaluation.

## Methods

All study procedures were performed in accordance with the protocol approved by the National Taiwan University Animal and Use Committee. Six-week-old male C57BL/6 J mice (*N* = 6, purchased from Laboratory Animal Center, National Taiwan University College of Medicine) were used for this study, and they were housed in the Animal Housing Facility of National Taiwan University, College of Life Science in polycarbonate cages with paddy husk bedding in the animal room. The room temperature and relative humidity were maintained at 21 ± 2 °C and 55% ± 20%, respectively, with a 12-h light/dark cycle.

### *Ganoderma lucidum* beta 1,3/1,6 glucan

Our previous study examined the safety of MBG [18], and in the present study, the mycelia of *G. lucidum* were subcultured and maintained in sterile yeast mould agar (0.02%) to produce MBG. The manufacturing process was initiated by preparing a culture medium containing glucose, lactose, galactose, sucrose, mannose, and yeast extract. The mycelia of *G. lucidum* were then introduced into the sterile medium and cultured using a shaker incubator at temperatures ranging from 27 °C to 32 °C for 3–5 weeks to achieve full polymerisation of MBG in the culture system. Subsequently, MBG from the cultured mycelia was homogenised and disrupted using a high-speed homogeniser and ultrasonic vibration. The MBG solution was then filtered and concentrated using a ceramic membrane to remove most of the residual small molecules in the solution. The concentrated MBG was dried through lyophilisation and then grinded into a powdered form. The sample contained approximately 95% carbohydrate, 1% fat, 1% protein, 2% ash, and 0.8% water. Using Megazyme (Ireland) mushroom and the Yeast Beta-Glucan Kit, we found that the crude extract contained approximately 60–65% MBG. The molecular weight of MBG was analysed through high-pressure liquid chromatography (HPLC) by using the Shodex sugar KS series containing KS-G, KS-804, and KS-805 columns and detected using a RI 2000 detector. The molecular weight was determined by referring to the standard curve by using standard molecules including STDP-800 (molecular weight 8 × 105), STDP-400 (molecular weight 4 × 105), STDP-200 (molecular weight 2 × 105), STDP-100 (molecular weight 1 × 105), and STDP-20 (molecular weight 2 × 104). The glycosyl linkage of MBG was also analysed. The sample was premethylated, depolymerised, reduced, and acetylated. The resultant partially methylated alditol acetates were then analysed through gas chromatography-mass spectrometry (GC–MS), according to the procedures described by York et al. and Ciucanu et al. [19, 20].

The results of HPLC showed that the MBG powder contained high-molecular-weight particles that ranged from 9.6 to 298 kDa, and the results of GC-MS showed that the MBG powder contained 2-; 4-; and 6-linked galactopyranosyl residues and 3-; 4; 3,4-; 2,4-; 4,6-; and 3,4,6-linked glucopyranosyl residues.

### Immunmodulation of the *G. lucidum* beta 1,3/1,6-glucan

#### Proliferation of spleen lymphocytes

The spleen cell proliferation was examined using 3-(4, 5-Dimethylthiazol-2-yl)-2, 5-diphenyltetrazolium bromide MTT assay to select the in vitro appropriate concentration of hydrogen peroxide functional role in the macrophage18-20. At the end of incubation, the cultured medium was removed and loading of 10 μl (5 mg/mL) MTT (SIGMA) into the plate and incubated at 27 °C for 4 h. Thereafter, 200 μL of dimethylsulfoxide (DMSO) was added to dissolve the formazan measured by microplate Spectrophotometer (μQunat, BioTek) at 590 nm.

The formula of Stimulation Ratio: (O.D. 590 in treatment/O.D. 590 in control)

#### NK cell-mediated cytotoxicity in mice

Mice were euthanized and the monocytes from the spleen were extracted for measurements of cytotoxicity. During the extraction procedure, spleens were removed and shredded with forceps, followed by separating the monocytes with centrifugation using Histopaque. The isolated monocytes were used as the effector cells after being washed twice with PBS buffer and had the cell density adjusted to 1 × 10^6^/mL in RPMI 1640 medium. YAC-1 cells, intended to be used as the target cells, were collected by centrifugation and had the cell density adjusted to 1 × 10^6^/mL. The cells were then stained with DiOC-18 at 37 °C, 5% CO_2_ for 20 min, followed by a PBS rinse, and suspended to 1 × 10^6^/mL in RPMI 1640 medium. For the assay of NK cell-mediated cytotoxicity, the effector and target cells were mixed in ratios of 10 : 1 followed by adding the propidium iodide (PI) staining solution to each mixture. Finally, the cell mixtures were incubated at 37 °C, 5% CO_2_ for 2 h, and analyzed with flow cytometer. Lysed (PI+ and DiOC-18+) and viable (DiOC-18+ and PI−) YAC-1 cells were identified by their dual- or single-positive staining. Assessment of the NK cell-mediated cytotoxicity was defined by the percentage increase in cytotoxicity relative to the baseline level set by the control group (100%).

#### Cytokines analysis

The IL-2 cytokines concentration of the blood was analysis by the ELISA method using the IL-2 ELISA Kit, Mouse (Thermo Fisher Scientific).

### Immunomodulation of inflammation

For examining G. *lucidum* beta 1,3/1,6-glucan as an immunomodulator in the mouse inflammatory response, the experimental group was divided into Group 1, ‘Control’, with oral feeding of twice-distilled water; Group 2, ‘Glucan’, with oral feeding of *G. lucidum* beta-glucan; and Group 3, ‘MIX’, with oral feeding of 200 μL of 2 × 10^9^ CFU/mL inactive Micrococcus lysodeikticus and 100 μL of 8 mg/mL *G. lucidum* beta-glucan.

#### Inactive *micrococcus lysodeikticus (M. lysodeikticus)*

*M. lysodeikticus* was cultured in brain heart infusion agar (Sigma) for 16 h and then washed with PBS. The *M. lysodeikticus* solution was dispensed into a 50-mL centrifuge tube with 10% formalin solution for 1 h and centrifuged at 1000 × g for 15 min to remove the supernatant. The centrifuged bacterial pellet was dissolved in 10 mL of PBS and centrifuged at 1000 × g for 15 min to completely remove the formalin.

#### Small intestine washing fluid

The small intestine was removed and 20 mL of PBS was irrigated through the intestinal lumen to obtain a small intestine washing fluid (SIWF) sample. The SIWF samples were centrifuged at 3000 rpm for 10 min, and the supernatant was collected and stored for analysis 17.

#### Serum

Blood was collected from the heart and stored at 4 °C for the serum sample.

#### Enzyme-linked immunosorbent assay

The concentrations of IgA and IgG were measured in SIWF and serum through ELISA (ICL, USA). In brief, separate 96-well plates were coated with the anti-mouse T IgA or IgG, 100 uL of SIWF and serum or a standard (ICL, USA) was added, and the plates were incubated at room temperature. The plates were washed five times, and a secondary antibody was added and incubated at room temperature for 1 h. After incubation, the plates were washed seven times, a substrate solution was added, and the plates were incubated in the dark. The reaction was stopped by a stop solution, and the absorbance was read using a microplate reader.

#### Real-time polymerase chain reaction

RT-qPCR was used to analyse the gene expression of poly-Ig receptor in the intestine, with β-actin used as the reference gene. The experiments were conducted by following the protocol published for SYBR Green Supermix Kits (Bio-Rad) and using Bio-Rad CFX384 touch RT-PCR for analysis (Table 1).

**Table 1:** Primer Sequences

Gene	Primer Sequences (5′ to 3′)	GeneBank No.
B-actin (Reference gene)	F: ACCACACCTTCTACAATGAG	BC138614.1
	R: ACGACCAGAGGCATACAG	
Poly-Ig receptor	F: AGGAGGTGAGTAGTATAGAAG	NM_011082.3
	R: GGAAGTTGATGAGGTTGG	

### Histopathological evaluations in mice fed a high-cholesterol diet

For examining *G. lucidum* beta-glucan as an immunomodulator in the mouse inflammatory response induced by a high-cholesterol diet, the experimental group was divided into Group 1,’Control’, with oral feeding of twice-distilled water; Group 2, ‘Glucan’, with oral feeding of *G. lucidum* beta-glucan; Group 3, ‘Cholesterol’, with a diet of 2% cholesterol with purified sodium cholate; and Group 4, ‘Glucan/Cholesterol’, with a diet of 2% cholesterol with purified sodium cholate and 100 uL of 8 mg/mL MBG, as presented in Table 2. The feed content (No. 47922291, TestDiet, USA) is presented in Table 3.

**Table 2:** Groups and Their Respective Treatments

Day	Feeding for 20 days	Day 21 (sacrifice)
Group		
Control (con)	twice-distilled water	×
Glucan (Glu)	100 ul of 8 mg/ml	×
Cholesterol (Cho)	2% cholesterol with sodium cholate purified Diet	×
Glucan/Cholesterol (Glu/Cho)	2% cholesterol with sodium cholate purified Diet and 100 ul of 8 mg/ml mushroom beta glucan	×

**Table 3:** Content of feed (No. 47922291, TestDiet, USA)

Ingredients	(%)
Dextrin	39.65
Casein-Vitamin Free	21
Sucrose	15
Lard	10
Mineral Mix	5
Powdered Cellulose	3
Sodium Cholate	2
RP Vitamin Mix	2
Cholesterol	2
Choline Chloride	0.2
DL-Methionine	0.15

#### Histopathological evaluation of tissues

The heart, liver, spleen, colon, and kidney were removed and fixed with neutralised and buffered formalin. Tissue sections were stained with Giemsa stain and observed using light microscopy. The eosinophilic granulocytes were stained light red, and the basophils and neutrophils were stained purple and blue, respectively. The Nile blue stain was used for staining lipids, and lipofuscin was stained blue.

##### Statistical analysis

The experimental data of each treatment group were divided by those of control group. Tukey’s honestly significant difference test and one-way ANOVA were used to analyse the statistical significance of differences between the treatment and control groups. A *p* value less than 0.05 was considered statistically significant. The results were presented as means ± SEM.

## Results

### Immunomodulation of the *ganoderma lucidum* beta 1,3/1,6 glucan

**Fig. 1:**
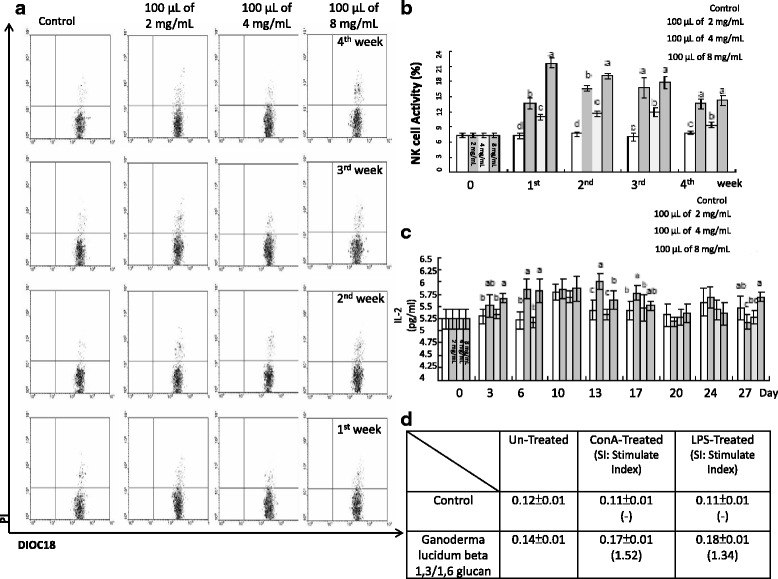
**a** Flow Cytometric profiles of NK Cell activity by FlowMax software. DiOC18+/PI+ defined as dead YAC-1, were represented as signals detected in the upper right-hand quadrant. **b** Effect of feeding with various concentration Ganoderma lucidum beta 1,3/1,6 glucan on the activity of NK cells. Values sharing a common superscript are significantly different (*p* < 0.05). **c** Concentration of IL-2 in mouse serum. Values sharing a common superscript are significantly different (*p* < 0.05). **d** Observation of spleen lymphocytes proliferation after Con-A or LPS stimulation

The NK cell-mediated cytotoxicity from healthy mice treated with Ganoderma lucidum beta 1,3/1,6 glucan was recorded. Results indicated that the cytotoxicity level increased significantly after treated with the Ganoderma lucidum beta 1,3/1,6 glucan, and with the level maintained through the days observation (Fig. 1a and b). In the serum cytokines, the experiment presented with a significant increasing trend through the 3^rd^ to 17^th^ days observation compared to the control (*p* <0.05), in the 20^th^ to the 27^th^ day observation, it was not with significant alteration (Fig. 1c). It was also recorded the spleen lymphocyte proliferation, in the research, it was with a little increase after treated with the *Ganoderma lucidum* beta 1,3/1,6 glucan (Fig. 1d).

### Immunoglobulin expression in small intestine washing fluid

**Fig. 2:**
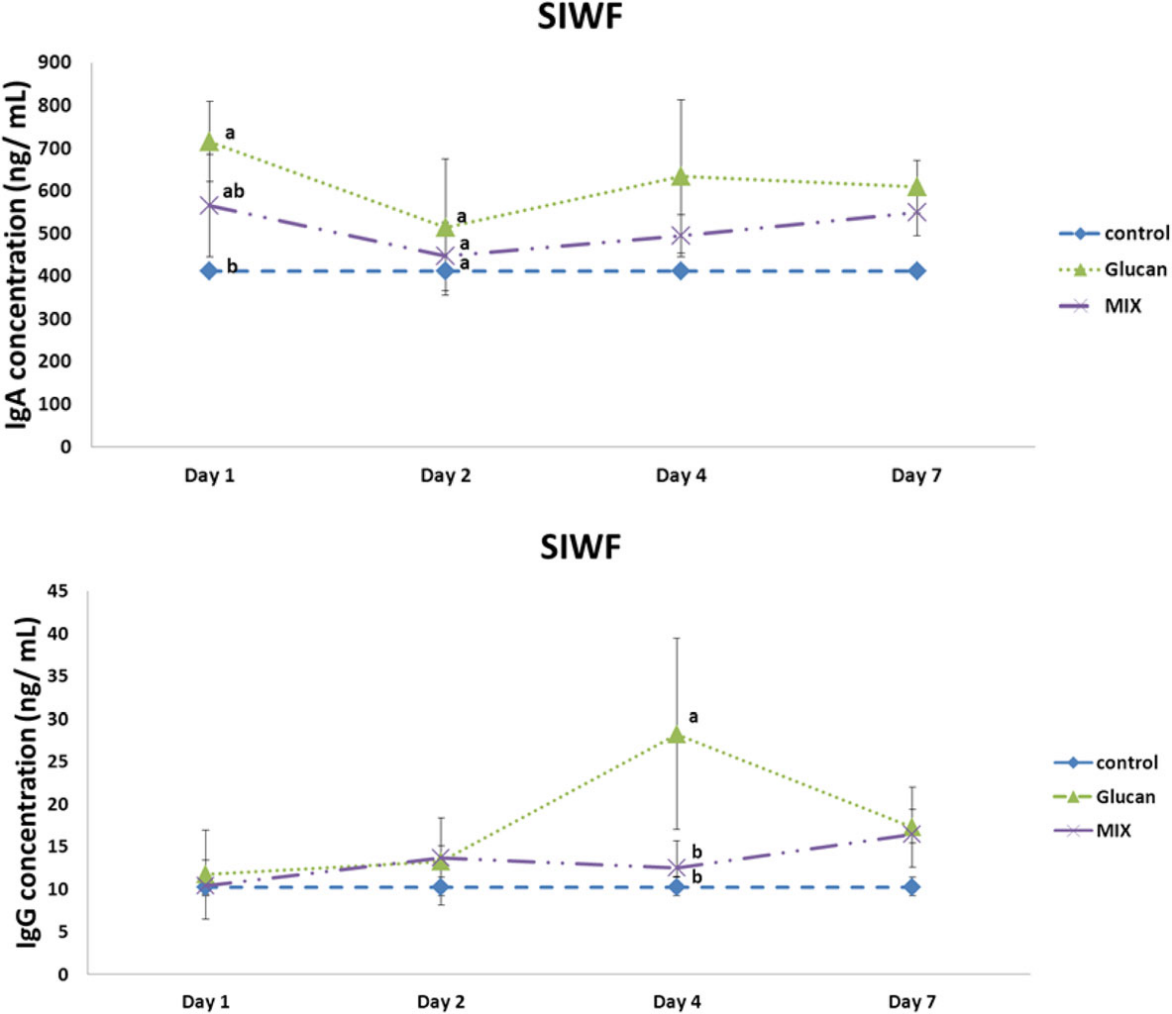
Production of immunoglobulin A and G in small intestine washing fluid on different days. Group 1, ‘Control’: oral feeding with twice-distilled water; Group 2, ‘Glucan’: oral feeding with *Ganoderma lucidum* beta-glucan; Group 3, ‘MIX’: oral feeding with 200 μL of 2 × 10^9^ CFU/mL inactive *Micrococcus lysodeikticus* and 100 μL of 8 mg/mL *G. lucidum* beta-glucan

IgA was the most abundant in the SIWF, with the average amount in the control SIWF being 412.23 ± 3.08 ng/mL. On the first day, the IgA concentration in the Glucan group was 715.21 ± 94.01 ng/mL higher than that in the Control and MIX (565.82 ± 120.22 ng/mL) groups. On the second day, there was no significant difference between the groups (*p* > 0.05), and the value in the Glucan and MIX groups was 514.60 ± 160.66 ng/mL and 445.97 ± 80.79 ng/mL, respectively. On the fourth day, IgA was at a concentration of 633.27 ± 178.99 ng/mL in the Glucan group and 494.29 ± 50.21 ng/mL in the MIX group, and there was no significant difference between the groups (*p* > 0.05). On the seventh day, the concentration of IgA was 609.17 ± 62.57 ng/mL in the Glucan group and 548.70 ± 53.51 ng/mL in the MIX group, and there was no significant difference between the groups. The average concentration of IgG produced in the SIWF was 10.15 ± 1.24 ng/mL. On the first day, the concentration of IgG was 11.69 ± 5.21 ng/mL in the Glucan group and 10.38 ± 3.03 ng/mL in the MIX group. On the second day, the concentration of IgG was 13.19 ± 5.12 ng/mL in the Glucan group and 13.63 ± 1.40 ng/mL in the MIX group. There was no significant difference between the groups (*p* > 0.05). On the fourth day, the concentration of IgG was 28.28 ± 11.24 ng/mL in the Glucan group, which was significantly higher than that in the MIX group (12.47 ± 3.14 ng/mL) (*p* < 0.05). On the seventh day, there was no significant difference between the groups, as illustrated in Fig. 2.

### Immunoglobulin expression in serum

**Fig. 3:**
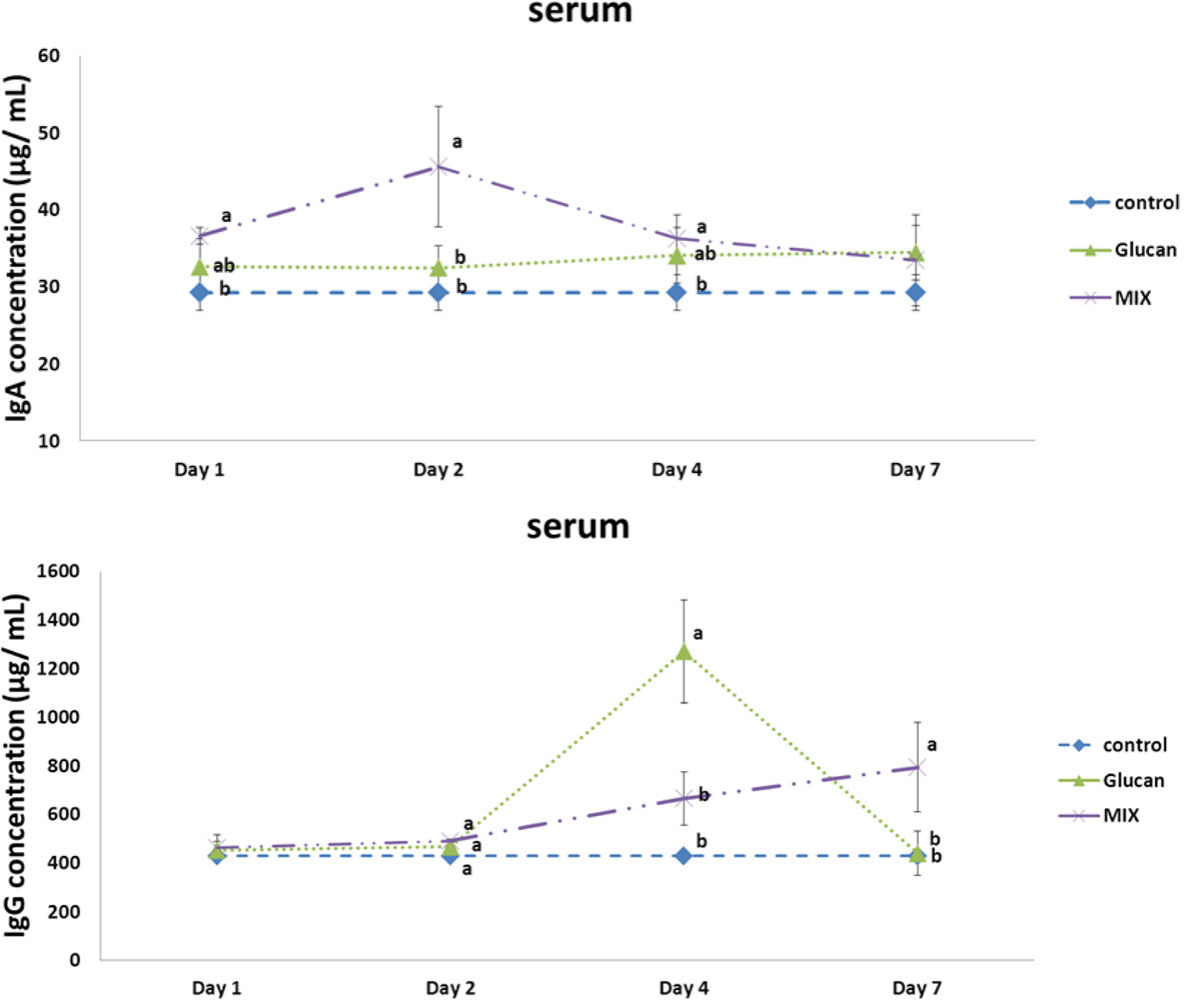
Immunoglobulin A and G production in the serum on different days. Group 1, ‘Control’: oral feeding with twice-distilled water; Group 2, ‘Glucan’: oral feeding with *Ganoderma lucidum* beta-glucan; Group 3, ‘MIX’: oral feeding with 200 μL of 2 × 10^9^ CFU/mL inactive *Micrococcus lysodeikticus* and 100 μL of 8 mg/mL *G. lucidum* beta-glucan

The average amount of IgA in the serum was 29.24 ± 2.29 μg/mL. On the first day, the concentration of IgA was 32.57 ± 3.64 μg/mL in the Glucan group and 36.60 ± 1.05 μg/mL in the MIX group, which were higher than that in the Control group (*p* < 0.05). On the second day, the concentration was 45.60 ± 7.87 μg/mL in the MIX group, which was higher than that in the Glucan group (32.47 ± 2.91 μg/mL) (*p* < 0.05). On the fourth day, the production of IgA was 36.30 ± 2.99 μg/mL in the MIX group, which was higher than that in the Glucan (34.03 ± 3.60 μg/mL) and Control groups (*p* < 0.05). On the seventh day, there was no significant difference between the groups (*p* > 0.05). The average production of IgG in the serum was 428.16 ± 22.25 μg/mL. On the first day, the concentration was 453.50 ± 36.37 μg/mL in the Glucan group and 462.78 ± 55.00 μg/mL in the MIX group, which were higher than that in the Control group, and there was no significant difference between the groups (*p* > 0.05). On the second day, the concentration was 491.50 ± 5.49 μg/mL in the MIX group and 466.95 ± 17.30 μg/mL in the Glucan group. On the fourth day, the concentration was 666.95 ± 109.77 μg/mL in the MIX group, which was lower than that in the Glucan (1270.93 ± 211.01 μg/mL) and Control groups (*p* < 0.05). On the seventh day, the concentration in the MIX group was 794.72 ± 185.19 μg/mL, which was significantly higher than that in the Glucan group (439.85 ± 92.41 μg/mL), as illustrated in Fig. 3.

### Poly-Ig receptor relative gene expression in the intestine

**Fig. 4:**
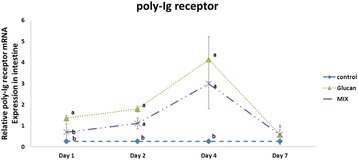
Poly-Ig Receptor relative gene expression in the intestine. Group 1, ‘Control’: oral feeding with twice-distilled water; Group 2, ‘Glucan’: oral feeding with *Ganoderma lucidum* beta-glucan; Group 3, ‘MIX’: oral feeding with 200 μL of 2 × 10^9^ CFU/mL inactive *Micrococcus lysodeikticus* and 100 μL of 8 mg/mL *G. lucidum* beta-glucan

In this research, the average poly-Ig receptor relative gene expression in the Control group was 0.26 ± 0.004. On the first day, the relative gene expression in the Glucan group was 1.36 ± 0.14, which was significantly higher than that in the MIX group, 0.70 ± 0.390 (*p* < 0.05). On the second day, the value was 1.80 ± 0.14 in the Glucan group and 1.11 ± 0.27 in the MIX group. On the fourth day, it was 4.13 ± 1.07 in the Glucan group and 2.98 ± 1.17 in the MIX group. The relative gene expression levels on the second and fourth days for the Glucan and MIX groups were both significantly higher than that in the Control group (*p* < 0.05), but were not different from each other. On the seventh day, there was no significant difference between the groups (*p* > 0.05), as illustrated in Fig. 4.

### Histopathological evaluation of tissues

**Fig. 5:**
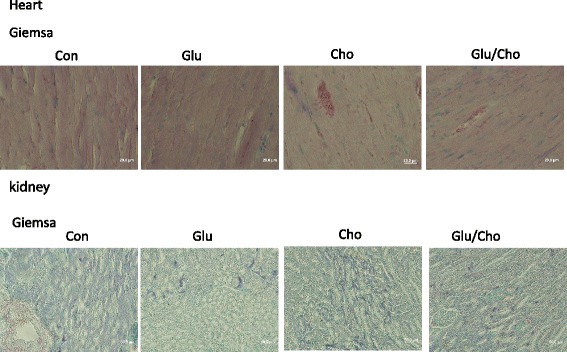
Histopathological evaluation of the heart and kidney using Giemsa stain. Group 1, ‘Control’: oral feeding with twice-distilled water; Group 2, ‘Glucan’: oral feeding with *Ganoderma lucidum* beta-glucan; Group 3, ‘Cholesterol’: a diet of 2% cholesterol with purified sodium cholate; Group 4, ‘Glucan/Cholesterol’: a diet of 2% cholesterol with purified sodium cholate and 100 μL of 8 mg/mL mushroom beta-glucan

The results illustrated in Fig. 5 indicate that the expression of basophilic granulocytes (purple) and neutrophils (light blue) was significantly higher in the Cholesterol group than in the other groups. In the Glucan/Cholesterol group, inflammatory cell expression in the heart tissue was significantly reduced compared with that in the Cholesterol group according to Giemsa staining. In the kidney, the expression of basophilic granulocytes (purple) and neutrophils (light blue) was significantly increased in the Cholesterol group. In the Glucan/Cholesterol group, a significantly reduced number of inflammatory cells was observed compared with that in the Cholesterol group.

**Fig. 6:**
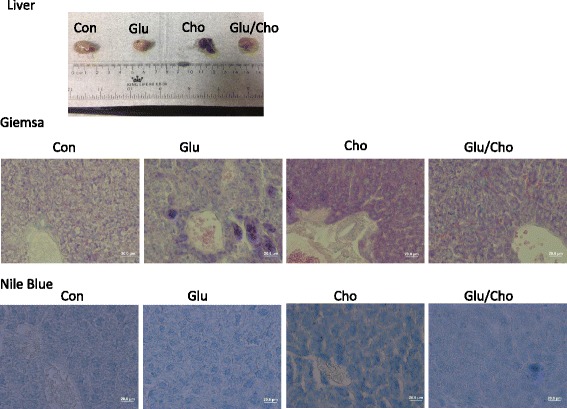
Histopathological evaluation of the liver using Giemsa and Nile blue stains. Group 1, ‘Control’: oral feeding with twice-distilled water; Group 2, ‘Glucan’: oral feeding with *Ganoderma lucidum* beta-glucan; Group 3, ‘Cholesterol’: a diet of 2% cholesterol with purified sodium cholate; Group 4, ‘Glucan/Cholesterol’: a diet of 2% cholesterol with purified sodium cholate and 100 μL of 8 mg/mL mushroom beta-glucan

**Fig. 7:**
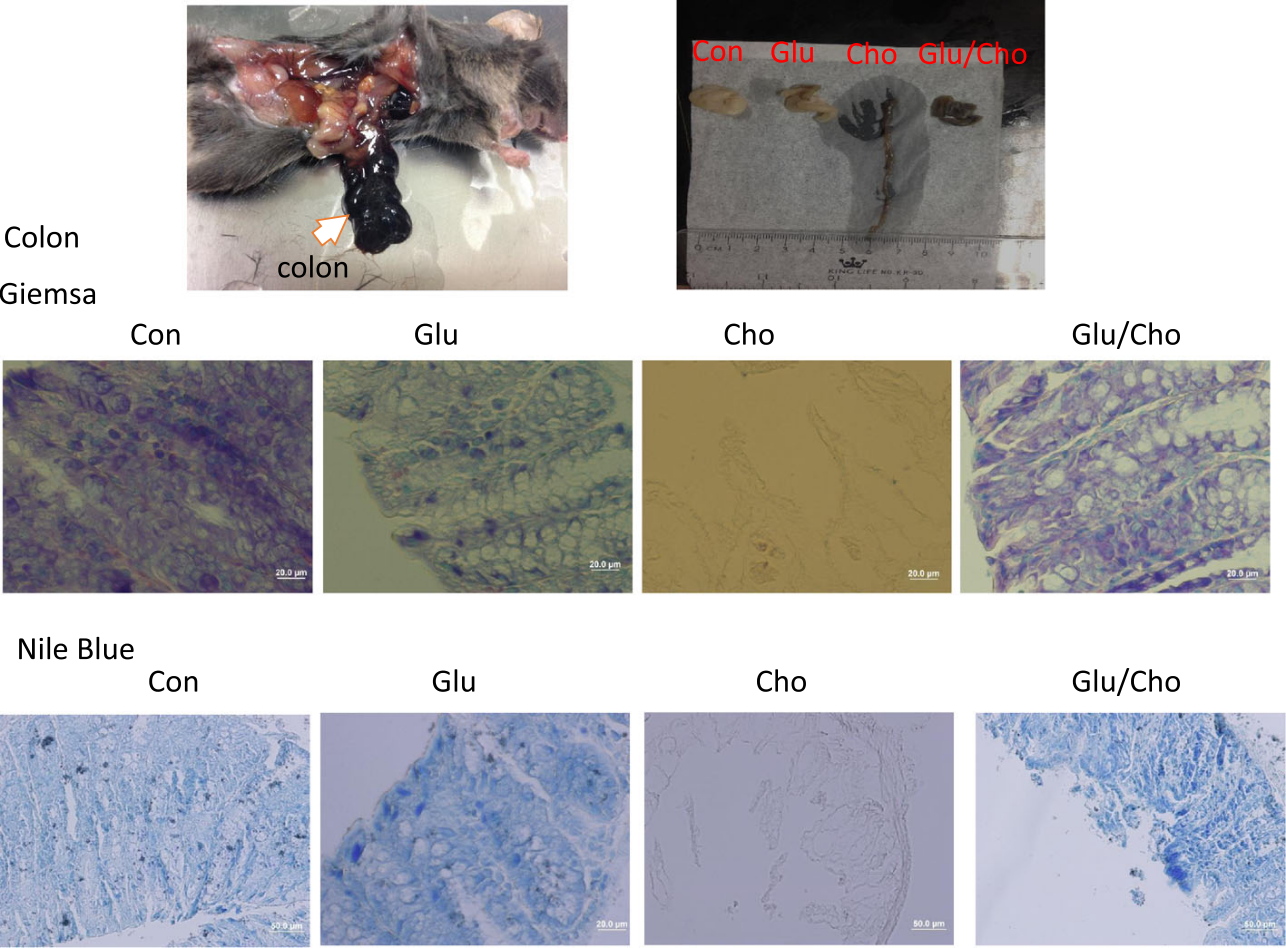
Histopathological evaluation of the colon using Giemsa and Nile blue stains. Group 1, ‘Control’: oral feeding with twice-distilled water; Group 2, ‘Glucan’: oral feeding with *Ganoderma lucidum* beta-glucan; Group 3, ‘Cholesterol’: a diet of 2v cholesterol with purified sodium cholate; Group 4, ‘Glucan/Cholesterol’: a diet of 2% cholesterol with purified sodium cholate and 100 μL of 8 mg/mL mushroom beta-glucan

**Fig. 8:**
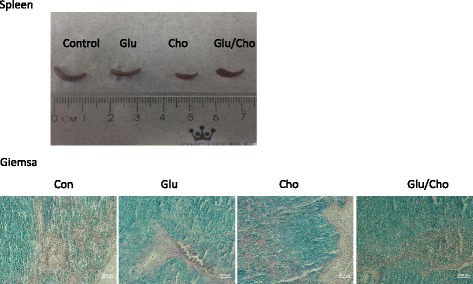
Histopathological evaluation of the spleen using Giemsa stain. Group 1, ‘Control’: oral feeding with twice-distilled water; Group 2, ‘Glucan’: oral feeding with *Ganoderma lucidum* beta-glucan; Group 3, ‘Cholesterol’: a diet of 2% cholesterol with purified sodium cholate; Group 4, ‘Glucan/Cholesterol’: a diet of 2% cholesterol with purified sodium cholate and 100 μL of 8 mg/mL mushroom beta-glucan

The histopathological evaluation of the liver revealed that the expression of eosinophilic granulocytes (red) and neutrophils (light blue) was higher in the Cholesterol group compared with the other groups. In the Glucan/Cholesterol group, the image revealed a reduced number of inflammatory cells compared with the Cholesterol group according to Giemsa staining. The Nile blue stain was used to observe lipid accumulation in the tissue, and the accumulation of lipoid and lipofuscin (blue) was increased in the Cholesterol group compared with the other groups. In the Glucan/Cholesterol group, the accumulation of lipoid and lipofuscin was significantly reduced, as illustrated in Fig. 6. The colon tissue appeared dark black in the Cholesterol group. In addition, according to Giemsa and Nile blue staining, the colon tissue appeared severely necrotic and eroded in the Cholesterol group. In the Glucan/Cholesterol group, the colon tissue was more complete compared with that in the Cholesterol group. Under the light microscope, the colon tissue showed accumulation of inflammatory cells, lipoid, and lipofuscin, but exhibited complete patterns, as illustrated in Fig. 7. The spleen tissue after Giemsa staining showed an increased number of eosinophilic and neutrophil granulocytes in the Cholesterol group compared with the other groups, and the number of inflammatory cells was reduced in the Glucan/Cholesterol group, as illustrated in Fig. 8.

## Discussion and conclusions

Mice fed a high-cholesterol diet were observed to realise whether the diet induced a pathological response in the liver. The results of this research indicated that the liver of C57BL/6 J mice exhibited severe inflammatory characteristics. In addition, the high-cholesterol diet increased expression of the transcription factor NF-κB relative to that in the Control group and induced the infiltration of inflammatory macrophages into the tissue in the short term. Moreover, a previous study demonstrated that a decreased fat intake may reduce very-low-density lipoprotein production in the plasma and prevent the development of liver inflammation and live foamy cells [21].

Mushroom beta 1,3–1,6 glucan is a complex, high-molecular-weight polysaccharide that is present in the cell wall of various mushrooms and has obvious immunomodulatory functions [22, 23]. Mushroom beta 1,3–1,6 glucan has a variety of effects in animals with immunomodulatory functions such as raising the phagocytic activity of macrophages and increasing the number of natural killer cells to enhance the ability to stimulate cytokines and thus activate the immune system. Macrophages, dendritic cells, and other nonimmune cells comprise nonspecific pattern-recognition receptors for complement receptor type 3 (CR3 receptor), which is a well-known beta-glucan receptor [24]. A study demonstrated that MBG combining with the CR3 receptor leads to a series of signal transfers (signalling cascades), which activates the transcription factors for regulating the inflammation response, the antigen-presenting procedure, and major histocompatibility complex performance [25].

A correlation study reported that feeding mice a high-cholesterol diet led to a high number of eosinophils, an increase in IL-5, and high concentrations of PGE2 and MCP-1 in the lung tissue. In an ex vivo experiment, cultured lymphocytes released high concentrations of IL-4 and IFN-γ; moreover, these inflammatory factors were significantly and positively correlated with serum cholesterol concentrations [26, 27]. Adding drinking water with a cholesterol-lowering drug, pravastatin, significantly reduced the infiltration of eosinophils and the expression of IL-5, PGE2, and MCP-1 in lung lavage fluid [27, 28].

The effect of a high-cholesterol diet in the inflammatory response was observed in heart, liver, kidney, spleen, and colon tissues through histopathological evaluations. The presented evidence demonstrates that the inflammation response in the high-cholesterol diet group was much higher than that in the other groups, particularly in the colon tissue, which exhibited necrosis and erosion.

Treatment of *Autrodia comphorata*-beta-glucan has been demonstrated to slow tumor growth and reduce the rate of metastasis [29] and illustrated that cytotoxic T-cells activity and tumor occurrence rate were observed. Results also showed that daily oral with *Grifola frondosua*-beta glucan is capable of enhancing the cytotoxic T-cells activity and decrease tumor occurrence rate [30]. Additionally, they found that the addition of conditioned medium with tumor cells into the progenitor cells of dendritic cells can further inhibit maturation of dendritic cells and lower the antigen presenting capability of the dendritic cells [31].

The immunotherapy is being developed with some beneficial advantage of alternative medicine as immunomodulation factors as mushroom beta glucan, antimicrobial peptides and the triterpenoid that represent a novel therapeutic approach for anti-inflammation as immunotherapy be an alternative therapy applied in the early phase of clinical therapy and immunomodulation on the early phase of immune disease.

Although *G. lucidum* beta 1,3/1,6-glucan acts as an immunomodulator and reduces the inflammatory response, the evidence does not directly support reduction in the accumulation of cholesterol in the organs.

## Abbreviations

*Ganoderma lucidum* beta 1,3/1,6: *G. lucidum* beta 1,3/1,6-glucan
